# Design and validation of a recognition instrument—the stroke aid for emergency scale—to predict large vessel occlusion stroke

**DOI:** 10.18632/aging.202910

**Published:** 2021-04-26

**Authors:** Baorui Zhang, Xiaochuan Huo, Fei Yuan, Guangrong Song, Lang Liu, Gaoting Ma, Dapeng Mo, Zhongxue Wu, Zhongrong Miao, Aihua Liu

**Affiliations:** 1 Beijing Neurosurgical Institute, Capital Medical University, Beijing 100070, China; 2 Department of Interventional Neuroradiology, Beijing Tiantan Hospital, Capital Medical University, Beijing 100070, China; 3 China National Clinical Research Center for Neurological Diseases, Beijing 100070, China; 4 Department of Neurosurgery, The Third Xiangya Hospital, Central South University, Changsha 410011, Hunan, China

**Keywords:** accuracy, endovascular therapy, large-vessel occlusion stroke, NIHSS, recognition instrument

## Abstract

Background and purpose: Rapidly recognizing patients with large-vessel occlusion stroke (LVOS) and transferring them to a center offering recanalization therapy is crucial of maximizing the benefits of early treatment. We therefore aimed to design an easy-to-use recognition instrument for identifying LVOS.

Methods: Prospective data were collected from emergency departments of 12 stroke-center hospitals in China during a 17-month study period. The Stroke Aid for Emergency (SAFE) scale is based on consciousness commands, facial palsy, gaze, and arm motor ability. Receiver operating characteristic analysis was used to obtain the area under the curve for the SAFE scale and previously established scales to predict LVOS.

Results: The SAFE scale could accurately predict LVOS at an accuracy rate comparable to that of the National Institutes of Health Stroke Scale (c-statistics: 0.823 versus 0.831, p = 0.4798). The sensitivity, specificity, positive predictive value, and negative predictive value for the SAFE scale were 0.6875, 0.8577, 0.6937, and 0.8542, respectively, with a cutoff point of 4. The SAFE scale also performed well in a subgroup analysis based on the patients’ ages, occluded vessel locations, and the onset-to-door times.

Conclusions: The SAFE scale can accurately recognize LVOS at a rate comparable to those of other, similar scales.

## INTRODUCTION

The combination of systemic thrombolysis and mechanical thrombectomy is highly effective for treating patients with large-vessel occlusion stroke (LVOS) [1, 2]. Because of the time-dependent effects of recanalization therapy, however, it is critical to recognize LVOS patients early and transfer them to the nearest comprehensive stroke center for maximizing early treatment benefits. It has been reported that each minute saved in the onset-to-door framework provides the patient, on average, with an additional 4.2 days of healthy life and increases the opportunity for successful vascular recanalization by 2.5% [3, 4].

The National Institutes of Health Stroke Scale (NIHSS) is originally designed to assess the severity of stroke patients and so used to identify LVOS, but it is complex and time consuming for Emergency Medical Services (EMS) despite good discriminative ability. Currently, some assessment tools simplify the NIHSS items, such as the Rapid Arterial Occlusion Assessment (RACE) Scale [5], Cincinnati Prehospital Stroke Severity Scale (CPSSS) [6], Los Angeles Motor Scale (LAMS) [7], Field Assessment Stroke Triage for Emergency Destination (FAST-ED) scale [8], and Three-Item Stroke Scale (3I-SS) [9], among others, to identify patients with LVOS. Although various scales have been designed, it is not clear which performs best in clinical practice. Thus, there is an urgent need to validate the scales to identify LVOS patients. Considering the limited availability of prehospitalization examinations and the time window effect of recanalization therapy, it is important to accurately identify patients with high likelihood of LVOS in the prehospital setting.

Few LVOS predicting scales are built based on Asian populations. In Asian population, the predominant reason for ischemic stroke is intracranial atherosclerosis (ICAS) which is quite different from Caucasian population that have a high rate of extracranial large artery atherosclerosis. In the Chinese population, ICAS is estimated to account for 33% to 50% of acute ischemic stroke [10]. This part of patients may have different functional manifestations when the stroke occurs. A scoring scale suitable for Asian population needs to be designed.

We, therefore, designed and validated an easy-to-perform, practical recognition instrument that we called the Stroke Aid for Emergency (SAFE) scale. We then compared it to other existing scales in regard of its ability to detect LVOS.

## MATERIALS AND METHODS

The data we analyzed came from a national multicenter registry study in China supported by the National Key Research and Development Program to assess key techniques and process improvements in reperfusion therapy for acute ischemic stroke. This project aimed to improve the key endovascular therapy (EVT) technology in stroke centers, standardize the emergency procedures of stroke centers to manage acute LVOS, and improve the standardization of emergency management of stroke in each center. The program was implemented through internet distance education, on-site expert guidance, nationwide teaching, and other methods to standardize the key technologies and management procedures of acute LVOS. Based on the national medical quality control platform, regular quality control was carried out for each central case.

During the implementation of the project, each participating center was equipped with the “Stroke First Aid” APP system of emergency treatment process management. The “Stroke First Aid” APP recorded patients' baseline information, imagine approach, treatment approach, stroke onset time, time of image examination, time of puncture, etc., in order to assess the stroke emergency treatment standardization of each participating center. We included ischemic stroke (stroke, transient ischemic attack, or stroke mimics) patients from 12 stroke centers in China between September 2017 and February 2019 in this study.

**Figure 1:**
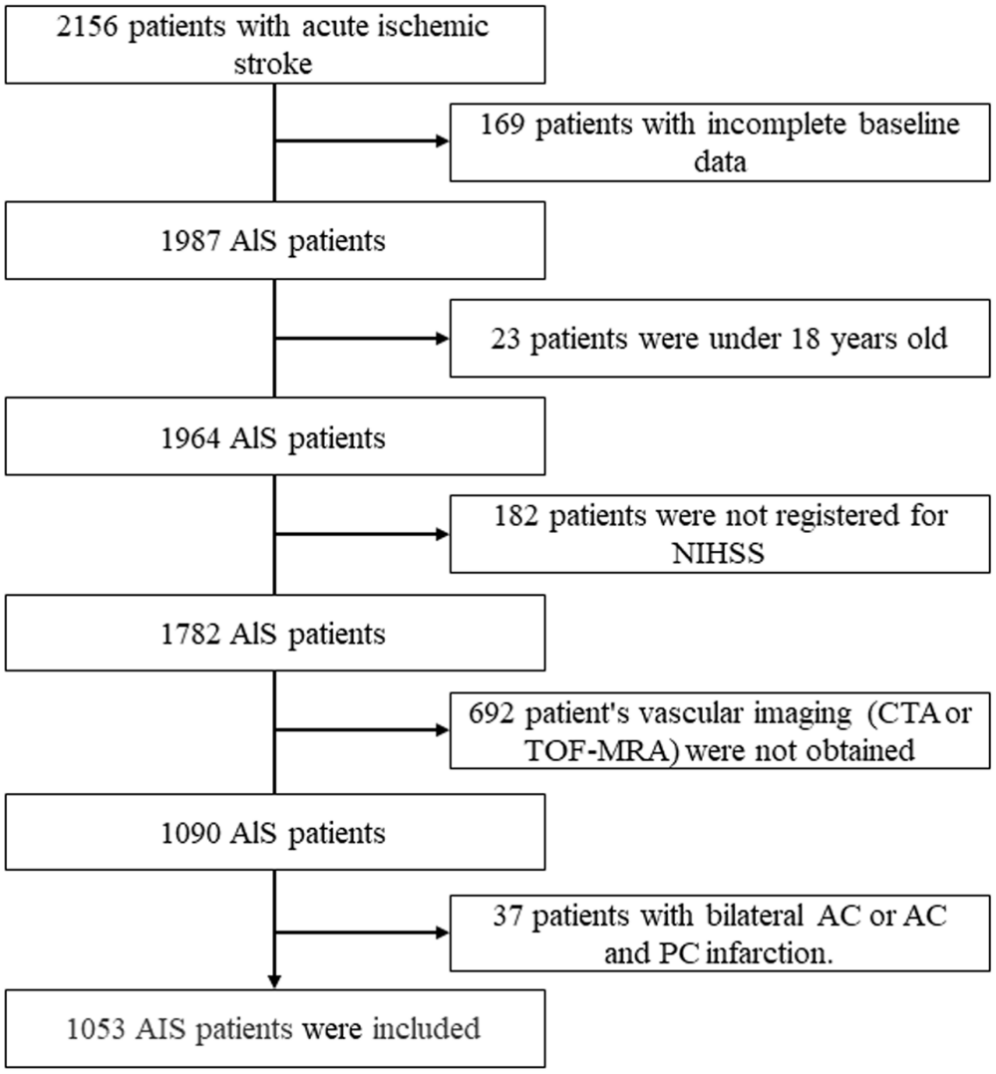
**Flowchart of the study population.** AIS: acute ischemic stroke; NIHSS, National Institutes of Health Stroke Scale; CTA: CT angiography; TOF-MRA: time-of-flight magnetic resonance angiography; AC: anterior circulation; PC: posterior circulation.

The inclusion criterion was an intracranial vascular examination—magnetic resonance angiography (MRA)/computed tomography angiography (CTA)/digital subtraction angiography—that had been completed within 24 h of stroke onset and before commencement of intravenous thrombolysis or endovascular therapy. The exclusion criteria were (1) age <18 years; (2) incomplete patient baseline data; (3) patient not registered for NIHSS; (4) lack of vascular imaging (CTA) or time-of-flight MRA (TOF-MRA); (5) bilateral anterior circulation infarction or anterior and posterior circulation infarction (Figure 1).

Baseline data included the patient’s age, sex, diabetes mellitus history, hypertension history, blood pressure, blood glucose, heart rate, the occluded vessel, onset-to-door time, NIHSS scale, and treatment strategy. Experienced neurologists conducted neurological examinations and assigned the patients an NIHSS scale at admission.

The design of the SAFE scale required, first, analyzing the items of the NIHSS to identify those predicted to have LVOS. We used logistic regression analyses and clinical judgment to select significant items that had the highest discriminatory values in the LVOS and non-LVOS groups in the derivation data set of patients suspected to have LVOS. The items that were discriminatory for LVOS and that were easy to assess for clinical symptoms were preferred. Arm motor ability was assessed for both arms, with the score of the more severely affected side accepted as the final assessment score.

Finally, the scale consisted of four items, including consciousness questions, facial palsy, gaze, and arm motor ability. In accordance with the severity of the symptoms, each item was divided into three levels: not severe (0 points), moderately severe (1 point), and seriously severe (2 points). The SAFE scale is shown in Table 1.

**Table 1:** The SAFE scale and its correspondence to the NIHSS.

**SAFE items**	**Annotation**	**SAFE scale**	**NIHSS source**
Consciousness questions (N1b)	Both correct	0	0
	One correct	1	1
	Neither correct	2	2
Gaze (N2)	Normal	0	0
	Partial gaze palsy	1	1
	Total gaze palsy	2	2
Facial palsy (N4)	Absent	0	0
	Mild	1	1
	Moderate to severe	2	2-3
Motor arm (N5a/b)	No drift	0	0
	Drift before 10s	1	1
	Falls before 10s, no antigravity effort, or no movement	2	2-4

LVOS was defined as total occlusion when it involved the intracranial internal carotid artery, M1 and M2 segments of the middle cerebral artery, and the basilar artery on baseline CTA or TOF-MRA [11]. Neurologists and radiologists with certified vascular and radiological experience assessed the occlusion via CTA or TOF-MRA, respectively, until agreement was reached.

The institutional review boards at all participating institutions approved this study. The study conformed with the tenets of the Declaration of Helsinki.

### Statistical methods

The entire data set was randomly divided into two subsets: a derivation data set (702 patients, 66.6%) and a verification data set (351 patients, 33.3%). For continuous variables, the differences between the groups were tested using Student’s t test or the Mann–Whitney U test. For categorical variables, a χ^2^ test was used to evaluate the differences between groups. Logistic regression analyses were performed on the NIHSS items to identify those that were significantly different between LVOS and non-LVOS in the derivation data set. The optimal cutoff of the SAFE scale was determined at the maximum Youden Index [12]. Sensitivity, specificity, positive predictive value, negative predictive value, Youden index, and overall accuracy were calculated regarding the ability to predict LVOS. Areas under the receiver operating characteristic curves (c-statistics) were compared to evaluate the predictive ability of each scale using the DeLong method [13].

All p values were based on two-sided tests, and p<0.05 was considered to indicate significance. All statistical analyses were carried out using R software (version 3.5.2; R Foundation for Statistical Computing, Vienna, Austria) and MedCalc Statistical Software version 19.0.7 (MedCalc Software Bvba, Ostend, Belgium; https://www.medcalc.org; 2019).

## RESULTS

Altogether, 2156 patients with ischemic stroke were prospectively enrolled, and 1103 patients were excluded in accordance with the exclusion criteria, leaving 1053 eligible patients, 364 (34.6%) of whom were women (Figure 1). Their median age was 65 years [interquartile range (IQR) 56–74 years]. The median score for the NIHSS was 6 (IQR 3–12), whereas the SAFE scale had a median score of 2 (IQR 0–4). The median onset-to-door time was 180 min (IQR 100–330 min). In all, 80.3% had an anterior circulation infarction, and 32.4% were diagnosed with LVOS. Among the patients, 69.1% underwent intravenous thrombolysis (IVT), and 30.9% underwent EVT. The baseline variables between LVOS and non-LVOS are shown in Table 2.

**Table 2:** Baseline variables between LVOS and non-LVOS patients in this study.

**Items**	**LVOS (n = 341)**	**Non-LVOS (n =712)**	**p value**
Age (years), median (IQR)	66(58-75)	64(55-74)	0.209
Male (n, %)	217(63.6)	472(66.3)	0.396
Diabetes Mellitus (n, %)	58(17.0)	109(15.3)	0.480
Hypertension (n, %)	275(80.6)	577(80.8)	0.879
Weight (kg), median (IQR)	65(56-73)	65(58-75)	0.264
Heart rate (bpm), median (IQR)	77(69-87)	80(70-89)	0.395
SBP (mmHg), median (IQR)	147(135-167)	151(138-166)	0.187
DBP (mmHg), median (IQR)	81(73-91)	84(74-100)	0.023
Glucose (mmol/L), median (IQR)	7.04 (5.78-9.19)	7.03 (5.90-8.60)	0.481
OTD time (min), median (IQR)	241 (150-420)	146 (85-268)	<0.001
NIHSS, median (IQR)	15(8-20)	4(2-8)	<0.001
CPSSS, median (IQR)	2(1-4)	0(0-1)	<0.001
3I-SS, median (IQR)	4(2-4)	1(0-2)	<0.001
RACE, median (IQR)	6(4-7)	1(0-4)	<0.001
FAST-ED, median (IQR)	4(2-5)	1(0-3)	<0.001
SAFE, median (IQR)	4(3-6)	1(0-3)	<0.001
Anterior circulation infarction (n, %)	252 (73.9)	594 (83.4)	<0.001
Arterial Imaging (n, %)			
CTA	29 (9.1)	49 (3.5)	0.621
MRA	72 (17.6)	158 (20.4)	
MRA+ CTA	240 (66.6)	505 (68.3)	
Treatment strategy (n, %)			
IVT	30(8.8)	698(98.0)	<0.001
EVT	311(91.2)	14(2.0)	
Occlusion Vessel (n, %)			
ICA	67(19.6)		
ICA +MCA	48(14.1)		
M1-2	137(40.2)		
BA	89(26.1)		

SBP, systolic pressure; DBP, diastolic pressure; OTD, onset-to-door time; IVT, Intravenous thrombolysis; EVT, Endovascular treatment; ICA, internal carotid artery; MCA, middle cerebral artery; BA, basilar artery.

Supplementary Table 1 shows the baseline variables for LVOS and non-LVOS in the derivation data set. The median age was 65 years (IQR 55–74 years), and 243 (34.6%) were women. The median onset-to-door time was 180 min (IQR 100–340 min). The patients with LVOS had higher NIHSS than the non-LVOS patients, had more anterior circulation infarctions, and a higher proportion underwent EVT. Data distributions for the verification data set are shown in Supplementary Table 2.

NIHSS items and its odds ratios regarding LVOS are shown in Supplementary Table 3. Consciousness questions, best gaze, facial palsy, and left and right arm weakness were independently associated with LVOS.

Table 3 shows the cutoff values for the SAFE scale for detecting LVOS in the derivation data set patients. The SAFE scale was calculated on the basis of the NIHSS and showed a predictive value similar to that of the NIHSS for detecting LVOS (c-statistics: 0.845 versus 0.850, p = 0.5212). A SAFE scale of ≥4 had a sensitivity of 0.8647, specificity of 0.6987, positive predictive value of 0.7143, negative predictive value of 0.8556, and accuracy of 0.8105 for detecting LVOS.

**Table 3:** Diagnostic test parameters of each SAFE scale threshold in derivation data set.

**Threshold**	**Sensitivity**	**Specificity**	**PPV**	**NPV**	**Youden**	**Accuracy**
SAFE≥8	0.0699	0.9873	0.7273	0.6869	0.0572	0.6880
SAFE≥7	0.1223	0.9810	0.7568	0.6977	0.1032	0.7009
SAFE≥6	0.3057	0.9577	0.7778	0.7402	0.2634	0.7450
SAFE≥5	0.4760	0.9260	0.7569	0.7849	0.4020	0.7792
SAFE≥4	0.6987	0.8647	0.7143	0.8556	0.5634	0.8105
SAFE≥3	0.7642	0.7865	0.6341	0.8732	0.5507	0.7792
SAFE≥2	0.8996	0.6110	0.5282	0.9263	0.5106	0.7051
SAFE≥1	0.9651	0.3467	0.4170	0.9535	0.3118	0.5484

Thresholds of each scale for predicting LVOS were evaluated and compared with those of the verification database (Table 4). Areas under the receiver operating characteristic curves (c-statistics) of scales in the verification database are shown in Table 5. Subgroup analysis was performed on the basis of age, occluded vessel location, and time of onset.

**Table 4:** Thresholds of each scale for detecting LVOS according to sensitivity, specificity, PPV and NPV, and accuracy in verification data set.

**Scales**	**Sensitivity**	**Specificity**	**PPV**	**NPV**	**Youden**	**Accuracy**
NIHSS≥10	0.6964	0.8285	0.6555	0.8534	0.5249	0.7863
CPSSS≥2	0.5804	0.8619	0.6633	0.8142	0.4423	0.7721
RACE≥3	0.8393	0.6946	0.5629	0.9022	0.5339	0.7407
3I-SS≥3	0.5982	0.8828	0.7053	0.8242	0.4811	0.7920
FAST-ED≥3	0.7143	0.7782	0.6015	0.8532	0.4925	0.7578
SAFE≥4	0.6875	0.8577	0.6937	0.8542	0.5452	0.8034

PPV, positive predictive value; NPV, negative predictive value.

**Table 5:** Areas under ROC of scales for validation and subgroup analysis in verification data set.

	**NIHSS**	**CPSSS**	**RACE**	**3I-SS**	**FAST-ED**	**SAFE**
Total						
c-statistics	0.831	0.807	0.801	0.796	0.805	0.823
p value	reference	0.084	0.023	0.004	0.023	0.480
Subgroup analysis						
The patient was over 60 years old						
c-statistics	0.872	0.847	0.844	0.833	0.844	0.871
p value	reference	0.098	0.069	0.012	0.033	0.910
The patient was 60 years or younger						
c-statistics	0.749	0.721	0.719	0.725	0.726	0.722
p value	reference	0.361	0.256	0.298	0.336	0.262
Occluded vessels are located in anterior circulation						
c-statistics	0.828	0.817	0.809	0.789	0.810	0.827
p value	reference	0.421	0.211	0.009	0.169	0.926
Occluded vessels are located in posterior circulation						
c-statistics	0.841	0.788	0.782	0.811	0.801	0.813
p value	reference	0.166	0.046	0.261	0.185	0.273
Intracranial vessels were assessed within 1.5 hours of onset						
c-statistics	0.811	0.786	0.775	0.762	0.688	0.800
p value	reference	0.586	0.240	0.229	0.107	0.624
Intracranial vessels were assessed at 1.5 hours or more after onset						
c-statistics	0.836	0.805	0.805	0.785	0.825	0.830
p value	reference	0.058	0.050	<0.001	0.436	0.669
Intracranial vessels were assessed within 6 hours of onset						
c-statistics	0.839	0.807	0.795	0.786	0.814	0.814
p value	reference	0.058	0.011	0.002	0.089	0.077
Intracranial vessels were assessed at 6 hours or more after onset						
c-statistics	0.826	0.806	0.821	0.773	0.800	0.861
p value	reference	0.048	0.857	0.030	0.272	0.188

SAFE, Stroke Aid for Emergency scale; FAST-ED, Field Assessment Stroke Triage for Emergency Destination scale; NIHSS, National Institutes of Health Stroke Scale; RACE, Rapid Arterial Occlusion Evaluation Scale.

**Figure 2:**
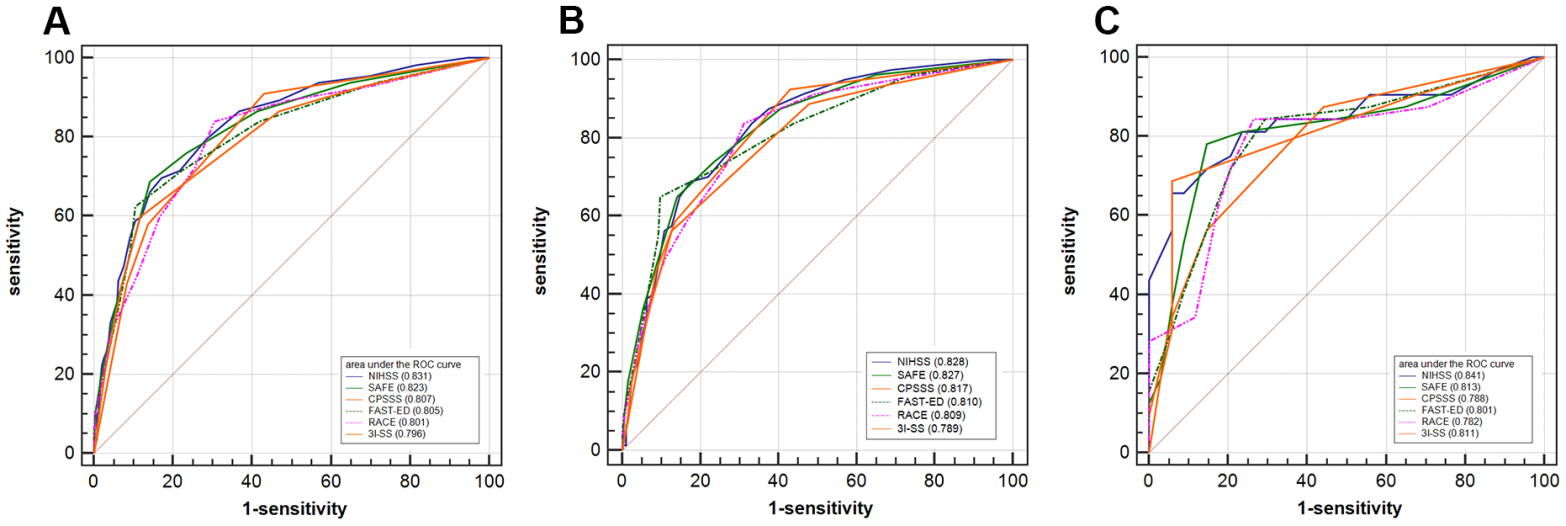
**Receiver operating characteristic curves comparing the discrimination of NIHSS, SAFE, CPSSS, FAST-ED, RACE, and 3I-SS.** (**A**) Receiver operating characteristic curves comparing the discrimination of SAFE, NIHSS, CPSSS, FAST-ED, RACE, and 3I-SS for the detection of large vessel occlusion strokes (all subjects). (**B**) Subjects with anterior circulation infarcts. (**C**) Subjects with posterior circulation infarcts. NIHSS: National Institutes of Health Stroke Scale; SAFE: Stroke Aid for Emergency Scale; CPSSS: Cincinnati Prehospital Stroke Severity Scale; FAST-ED: Field Assessment Stroke Triage for Emergency Destination scale; RACE: Rapid Arterial Occlusion Evaluation scale; 3I-SS: Three-Item Stroke Scale.

The receiver operating characteristics curve is compared with the NIHSS and other scales in Figure 2. The SAFE scale showed a predictive value similar to that of the NIHSS for detecting LVOS (c-statistics: 0.823 versus 0.831, p = 0.4798).

## DISCUSSION

We established a SAFE scale comprising four items including consciousness commands, facial palsy, gaze, and arm motor ability. The present study showed that the ability of the SAFE scale to identify LVOS was comparable to that of NIHSS and other, similar scales.

Disturbance of consciousness is one of the most frequent manifestations of stroke, and the assessment of the consciousness level is complicated [14]. Using the SAFE scale, we asked questions that were relatively modest and practicable to assess the level of consciousness disturbance. In addition, we considered that a combination of the consciousness level and some neurological sign (e.g., gaze bias) could reflect the extent of a cerebral cortex infarction after large-artery occlusion [15, 16]. Facial paralysis as a typical sign of LVOS in the anterior or posterior circulation and has been shown to be a NIHSS item with the best ability to distinguish stroke from mimicries [17]. Gaze bias also occurs when the para-middle pontine reticular structure (the pontine gaze center) is affected (also seen in the occlusion of the great arteries in the posterior circulation affecting the brainstem) [18]. Facial palsy and Gaze bias were included in the SAFE scale as they could reflect anterior and posterior circulation infarctions. This study included both anterior and posterior circulatory stroke, whereas some other LVOS scales were designed to focus only on anterior circulation.

Several scales have been designed to detect LVOS. Some authors considered that an NIHSS scale cutoff of 6 or 10 could predict LVOS in patients with acute ischemic stroke [19, 20]. The RACE scale has been applied in the field and has identified LVOS, but it was validated where 50% of the patients were diagnosed by transcranial Doppler ultrasonography, which was less sensitive and specific than CTA or TOF-MRA for detecting LVOS [5]. The RACE scale was designed based on data from anterior circulation stroke patients, reducing their application for patients with a posterior circulation stroke. FAST-ED scale has been widely used and shows the better sensitivity to the correct diagnosis of LVOS. However, its application in posterior circulation stroke is limited due to its insufficient posterior circulation stroke derivative cohort [8]. The derived cohort of the SAFE scale included both anterior and posterior circulation stroke, and the SAFE scale was relatively effective in predicting posterior circulation LVOS.

Studies have demonstrated that EVT treatment of vertebrobasilar artery occlusion stroke is not inferior to standard medical treatment [21]. The SAFE scale has relatively good performance for screening out posterior circulation LVOS, which provides a tool for immediate EVT of posterior circulation LVOS in emergency. Our study showed that the AUC value of SAFE scale for the recognition of posterior circulation LVOS was 0.813, which was similar to that of NIHSS (0.841) without significant difference (p=0.273). Compared with other scales, the specificity of the SAFE scale in this study was higher, which may be related to the exclusion of patients with intracranial hemorrhage in this study. Other cohorts with a higher proportion of intracranial hemorrhage reported lower specificity, possibly because intracranial hemorrhage patients had the highest rate of being evaluated as false positives [11].

The time window of EVT for treating acute LVOS has been extended to 24h after onset [22, 23], so a new scale that can predict LVOS within 24 hours after stroke is required. However, the derived cohort of other scales, such as RACE [5], CPSSS [6], and 3I-SS [9], are included stroke patients within 6 hours after stroke onset. Some studies aimed to verify the accuracy of the LVOS predictive scales have also set the stroke onset time to less than 6 hours. In this study, the derived cohort of SAFE scale, like the FAST-ED and ROSIER derived cohort [8, 24], included stroke patients within 24 hours after stroke onset. Thus, the SAFE scale is an extension of the application of the pre-hospital emergency scale.

The accuracy of the NIHSS to predict LVOS, however, is time-dependent, which reduces the value of the application for patients with longer onset times. Heldner et al. found that the ability of NIHSS to predict LVOS was most accurate during the early hours of symptom onset [25], similar to the results of the present study. The SAFE scale for predicting LVOS has relatively better accuracy during certain time periods after symptom onset. Although the accuracy of the SAFE scale for predicting LVOS is preferable during different time periods, we believe that an imaging examination should be performed as soon as possible to determine which intracranial vessels are occluded within a limited time. Finally, the SAFE scale can be used as a triage tool for the pretreatment examination.

This study of the new scale has some strengths. First, it is a multicenter study with ischemic stroke patients consecutively enrolled at 12 stroke centers across China. The results thus reflect the scale’s LVOS recognition ability in a varied Chinese population. Hence, the scale may be applicable to more varied Asian populations. Second, we studied all patients with CTA and TOF-MRA with external validation of the imaging. Third, this is a prospective cohort, and we compared the SAFE scale results with those obtained using other scales to further verify its accuracy in a subgroup analysis.

This study has some limitations. First, we compared the scales based on an extrapolation from the NIHSS. Although the treatment strategy depends on the patients’ symptoms at admission, future studies should assess the patients’ conditions via prehospitalization examinations, using the results to serve as a triage tool because symptoms can alter substantially during the early phase of stroke. Second, some patients with mild symptoms have LVOS that might have been missed when using this scale to screen for LVOS, in spite of that whether patients with LVOS and NIHSS scores of <6 points could benefit from EVT remains controversial. Third, we observed a 32.4% rate of LVOS in our cohort, higher than that of the general population, which may affect the extrapolation of the results. Fourth, we did not evaluate grip strength, so we cannot compare it with the LAMS. Furthermore, we could not evaluate the consistency of the emergency procedures of each participating unit, although all the stroke centers have been rigorously trained. Finally, our cohort consists only of patients with a confirmed acute ischemic stroke receiving IVT or EVT, and hemorrhagic stroke was ruled out by brain imaging. Consequently, sensitivity and specificity of the SAFE scale might differ in prehospital cohorts with suspected stroke that include hemorrhagic strokes. Further comprehensive and prospective studies with homogeneous populations are needed to test the SAFE scale with more robust, substantial results.

## CONCLUSIONS

The SAFE scale can recognize LVOS at a level of accuracy that is similar to those of other scales.

## Supplementary Material

Supplementary Tables
