# Supplementary material for: Design and validation of a recognition instrument—the stroke aid for emergency scale—to predict large vessel occlusion stroke
Source: Aging (Albany NY). 2021 Apr 26;13(10):13680–92. doi: 10.18632/aging.202910 (PMC8202851; doi:10.18632/aging.202910)
Supplement: Supplementary Tables [file aging-13-202910-s001.pdf]

SUPPLEMENTARY TABLES

Supplementary Table 1. Baseline variables between LVOS and non- LVOS in derivation data set.

Items	LVOs (n = 229)	Non-LVOs (n =473)	p value
Age (years), median (IQR)	66 (58-74)	64 (55-73)	0.314
Male (n, %)	145 (63.3)	314 (66.4)	0.423
Diabetes Mellitus (n, %)	38 (16.6)	67 (14.2)	0.398
Hypertension (n, %)	184 (80.3)	383 (81.0)	0.844
Weight (kg), median (IQR)	62 (56-73)	65 (57-75)	0.916
Heart rate (bpm), median (IQR)	77 (70-86)	80 (72-90)	0.253
SBP (mmHg), median (IQR)	146 (133-164)	152 (140-170)	0.093
DBP (mmHg), median (IQR)	83 (74-92)	84 (75-100)	0.083
Glucose (mmol/L), median (IQR)	6.60 (5.70-9.12)	7.10 (6.00-8.75)	0.705
OTD time (min), median (IQR)	246 (148-420)	149 (84-278)	<0.001
NIHSS, median (IQR)	14 (9-18)	4 (2-7)	<0.001
CPSSS, median (IQR)	2 (1-3)	0 (0-1)	<0.001
3I-SS, median (IQR)	3 (2-4)	1 (0-2)	<0.001
RACE, median (IQR)	5 (4-7)	1 (0-3)	<0.001
FAST-ED, median (IQR)	4 (2-5)	1 (0-2)	<0.001
SAFE, median (IQR)	4 (2-6)	1 (0-2)	<0.001
Anterior circulation infarction (n, %)	172 (75.1)	389 (82.2)	0.027
Arterial Imaging (n, %)			
CTA	20 (8.7)	31 (6.6)	0.574
MRA	49 (21.4)	106 (22.4)	
MRA+ CTA	160 (69.9)	336 (71.0)	
Treatment strategy (n, %)			
IVT	21 (9.2)	465 (98.3)	<0.001
EVT	208 (90.8)	8 (1.7)	
Occlusion Vessel (n, %)			
ICA	46 (20.1)		
ICA +MCA	32 (14.0)		
M1-2	94 (41.0)		
BA	57 (24.9)		

SBP, systolic pressure; DBP, diastolic pressure; OTD, onset-to-door time; IVT, Intravenous thrombolysis; EVT, Endovascular treatment; ICA, internal carotid artery; MCA, middle cerebral artery; BA, basilar artery.

Supplementary Table 2. Baseline variables between LVOS and non- LVOS in verification data set.

Items	LVOs (n =112)	Non-LVOs (n =239)	p value
Age (years), median (IQR)	66 (58-76)	65 (57-74)	0.426
Male (n, %)	72 (64.3)	158 (66.1)	0.738
Diabetes Mellitus (n, %)	20 (17.9)	42 (17.6)	0.948
Hypertension (n, %)	91 (81.3)	192 (80.3)	0.840
Weight (kg), median (IQR)	67 (56-76)	66 (60-71)	0.063
Heart rate (bpm), median (IQR)	75 (67-92)	76 (66-86)	0.761
SBP (mmHg), median (IQR)	152 (136-176)	148 (132-163)	0.826
DBP (mmHg), median (IQR)	81 (72-91)	86 (73-99)	0.158
Glucose (mmol/L), median (IQR)	7.30 (6.25-9.30)	6.80 (5.65-8.45)	0.082
OTD time (min), median (IQR)	240 (152-421)	145 (85-236)	<0.001
NIHSS, median (IQR)	14 (8-20)	4 (2-8)	<0.001
CPSSS, median (IQR)	2 (1-3)	0 (0-1)	<0.001
3I-SS, median (IQR)	3 (2-4)	1 (0-2)	<0.001
RACE, median (IQR)	5 (3-7)	1 (0-3)	<0.001
FAST-ED, median (IQR)	5 (2-6)	1 (0-2)	<0.001
SAFE, median (IQR)	4 (3-6)	1 (0-2)	<0.001
Anterior circulation infarction (n, %)	80 (71.4)	205 (85.8)	0.001
Arterial Imaging (n, %)			
CTA	9 (8.0)	18 (7.5)	0.959
MRA	23 (20.5)	52 (21.8)	
MRA+ CTA	80 (71.4)	169 (70.7)	
Treatment strategy (n, %)			
IVT	9 (8.0)	233 (97.5)	<0.001
EVT	103 (92.0)	6 (2.5)	
Occlusion Vessel (n, %)			
ICA	21 (18.8)		
ICA +MCA	16 (14.3)		
M1-2	43 (38.4)		
BA	32 (28.6)		

SBP, systolic pressure; DBP, diastolic pressure; OTD, onset-to-door time; IVT, Intravenous thrombolysis; EVT, Endovascular treatment; ICA, internal carotid artery; MCA, middle cerebral artery; BA, basilar artery.

Supplementary Table 3. NIHSS items and its odds ratios regarding LVOS.

Items	ORs	95% CI	P value
N1a. Level of consciousness	0.939	0.635-1.389	0.754
N1b. Questions	1.604	1.070-2.403	0.022
N1c. Commands	1.128	0.709-1.793	0.611
N2. Gaze	2.068	1.482-2.886	0.000
N3. Visual Fields	0.768	0.403-1.460	0.420
N4. Facial Palsy	1.286	1.006-1.643	0.045
N5a. Left motor Arm	1.537	1.187-1.991	0.001
N5b. Right motor Arm	1.371	1.030-1.825	0.031
N6a. Left motor Leg	1.058	0.795-1.408	0.698
N6b. Right motor Leg	1.138	0.851-1.524	0.383
N7. Ataxia	0.938	0.596-1.475	0.781
N8. Sensory	1.003	0.706-1.426	0.986
N9. Language	1.020	0.813-1.279	0.866
N10. Dysarthria	1.142	0.850-1.533	0.379
N11. Extinction/Neglect	1.142	0.624-2.092	0.666
